# Editorial for Special Issue “Yeasts Biochemistry and Biotechnology”

**DOI:** 10.3390/microorganisms12030534

**Published:** 2024-03-07

**Authors:** María-Isabel González-Siso, Manuel Becerra

**Affiliations:** Grupo EXPRELA (Regulación de la Expresión Génica y Aplicaciones), CICA—Centro Interdisciplinar de Química e Bioloxía, Facultade de Ciencias, Universidade da Coruña, 15071 A Coruña, Spain; manuel.becerra@udc.es

## 1. Introduction

Yeasts, both *Saccharomyces* and non-conventional strains, are currently the focus of active research due to their impressive applications in biotechnological bioprocesses, such as being hosts to produce recombinant proteins or main actors of the fermentation industries. Moreover, they are increasingly important tools in systems biology since they are used as simple models when studying the physiology or gene regulation of eukaryotic organisms.

## 2. An Overview of Published Articles

Ten papers, comprising two reviews and eight research papers, that represent recent developments in the above-mentioned fields have been published in this Special Issue. One of the reviews (Contribution 1) is a very nice description of the role that yeasts have played in history and their present applications in cancer research, with a focus on HMGB proteins.

The other review (Contribution 2) reports on the characteristics of non-*Sacharomyces* yeasts and their impact on wine fermentation, which contribute significantly to wine flavor and aroma and, therefore, consumer acceptance. 

Additionally, related to enology, Contribution 3 concerns beta-glucosidase from the yeast *Metschnikowia pulcherrima* (isolated from the surface of grapes). This enzyme shows suitable characteristics to be used in wine fermentations to liberate aromatic compounds.

Only one research paper of this Special Issue is devoted to *Saccharomyces cerevisiae* (Contribution 4), specifically to the purification and biochemical characterization of the enzyme P5C reductase that catalyzes the last step in both proline synthesis and arginine catabolism. 

Contributions 5 and 6 discuss the use of non-*Saccharomyces* yeasts in beer fermentations. The first contribution outlines brewing beers with zero or low ethanol content, and the second contribution discusses controlling Lambic beer fermentation. 

Two articles are related to using *Komagataella phaffi* (previously *Pichia pastoris*) as a protagonist. *K. phaffi* is a promising cell factory, a good producer of recombinant peptides and proteins, and it can use methanol and glycerol as carbon sources. To improve this biotechnological application, Contribution 7 studies the effect of *MPC1* gene deletion on lactic acid production, encoding a mitochondrial pyruvate carrier. Contribution 8 focuses on the effect of methionine on gene expression in a methanol-containing media. 

Similarly, Contribution 9 describes the identification of strong promoters and the construction of hybrid promoters to increase heterologous protein production in glycerol-based bioprocesses by another biotechnologically relevant yeast, *Yarrowia lipolytica*. Glycerol is an interesting substrate since it is the major byproduct of biodiesel production. 

Finally, with the objective of waste valorization, Contribution 10 reports on the use of carotenogenic yeasts of the genus *Rhodotorula* and *Sporidiobolus* for the processing of waste oils/fats and waste glycerol. The selected strains were able to metabolize substrates mixed with these components, yielding abundant biomass with a high carotenoid content.

## 3. Conclusions/Perspectives

Yeasts are the principal protagonists of biotechnological industries. Although *Saccharomyces cerevisiae* is considered “the yeast”, non-conventional yeasts have gained ground on *Saccharomyces* in recent years. This fact is reflected in the papers presented in this Special Issue. Overall, these papers show recent research advances in the use of yeasts for wine and beer fermentation, waste valorization, enzymes, and the production of recombinant proteins and carotenoids. There is still much to explore and improve concerning these topics that are of high social and economic relevance.

